# Supplementary material for: Parent reports of children’s emotional and behavioral problems in a low- and middle- income country (LMIC): An epidemiological study of Nepali schoolchildren
Source: PLoS One. 2021 Aug 3;16(8):e0255596. doi: 10.1371/journal.pone.0255596 (PMC8330921; doi:10.1371/journal.pone.0255596)
Supplement: Attachment — Submitted filename: Manuscript 1, PLOS ONE_R1.docx [file pone.0255596.s002.docx]

**Parent reports of child behavior problems in a low- and middle- income country (LMIC): An epidemiological study of Nepali schoolchildren**

Jasmine Ma^1,2*^, Pashupati Mahat^3^, Per Håkan Brøndbo^1^, Bjørn H. Handegård^1^, Siv Kvernmo^4^, Anne Cecilie Javo^1,5^

1. Regional Centre for Child and Youth Mental Health and Child Welfare -North, Faculty of Health Sciences, UiT The Arctic University of Norway, Tromsø, Norway
2. Child & Adolescent Psychiatry Clinic, Kanti Children’s Hospital, Kathmandu, Nepal
3. Centre for Mental Health and Counseling, Kathmandu, Nepal
4. Department of Clinical Medicine, Faculty of Health Sciences, UiT The Arctic University of Norway, Tromsø, Norway.
5. Sami National Competence Center for Mental Health, Sami Klinihkka, Finnmark Hospital Trust, Karasjok, Norway

*Corresponding author

Email: [jasminema2006@yahoo.com](mailto:jasminema2006@yahoo.com) (JM)

**Short title:**Parent reports of behavioral problems in Nepalese schoolchildren

**Abstract**

**Background:** As epidemiological data on child mental health in low- and middle-income countries are limited, a large-scale survey was undertaken to estimate the prevalence and amount of child emotional and behavior problems in Nepal.

**Methods:** 3820 schoolchildren aged 6-18 years were selected from 16 districts of the three geographical regions of Nepal, including rural, semi-urban and urban areas. We used the Nepali version of the Child Behavior Checklist (CBCL)/6-18 years as screening instrument. Comparisons of child problems between genders and between the seven largest castes and ethnic groups were carried out by analysis of variance. Prevalence was computed based on American norms.

**Results:** Adjusted prevalence of Total Problems was 18.3% (boys: 19.1%; girls:17.6%). The prevalence of internalizing problems was higher than externalizing problems. The means of Total, Externalizing, and Internalizing problems were 29.7 (SD 25.6), 7.7 (SD 8.0), and 9.1 (SD 8.1), respectively. The lowest Hindu caste (Khas Kaami) scored the highest, and the indigenous Tharu group scored the lowest on all scales. In the Mountains and Middle Hills regions, problem scores were higher in the rural areas, whereas, in the Tarai region, they were higher in the urban areas.

**Conclusion:** The prevalence and amount of emotional and behavioral problems in Nepalese children were found to be high compared to findings in meta-analyses worldwide. Problem scores varied according to gender, castes /ethnic groups, and living areas. Our findings highlight the need for a stronger focus on child mental problems in a low-and middle-income countries like Nepal.

**Introduction**

One third of the world’s population are children, with the vast majority living in low- and middle-income countries (LMIC) [1]. Despite documentation that child mental problems are a leading cause of health-related disability, mental health needs of children and adolescents are often neglected, especially in LMICs [2]. Epidemiological studies may enable the assessment of service needs as well as the identification of high-risk groups needing special attention.

However, studies on mental health problems in children and adolescents in LMICs are sparse. A recent review on the global coverage of prevalence data for mental disorders in children aged 5-17 years reported that most of the LMICs had no data on any kind of mental disorders. It further reported that many LMICs were poorly represented in the available data; for example, no region in sub-Saharan Africa had more than 2% coverage for any disorder [3]. Although sparse, previous research in LMICs suggests that child and adolescent mental health problems are common. A systematic review in non-referred samples from LMICs showed a prevalence of about 10–20% in most of the 16 surveys, which is consistent with findings from high income countries (HIC) [2].

A former meta-analysis of 51 Asian countries reported a general prevalence of 10-20% [4]. In the South Asian countries, studies on child mental health are sparse and their quality varies. In India, a review study involving both school-based and community-based studies, reported a prevalence of 23.3% in the school-based studies and 6.5% in the community-based studies. The discrepancy was probably due to several methodological and sample factors in the latter studies [5]. In a systematic review from Bangladesh, the prevalence of mental disorders was found to range from 13.4% to 22.9% among children aged 2-16 [6]. In Pakistan, a study among school children aged 6-16 reported a prevalence of 15.9% of behavior problems and 22.5% of emotional problems [7]. In another LMIC country, Iran, a community-based study of children aged 6-17 reported a prevalence rate of 16.7% of total difficulties [8]. In China, a recent, large-scale epidemiological study in the Sichuan Province reported an overall prevalence of 19.1% of child mental disorders [9].

For Nepal, we do not know the magnitude of EBP in the child population as no larger studies have been published internationally. However, an unpublished Nepali PhD dissertation from 2007 on teacher-reported problems of children aged 6-18, suggested a prevalence rate of 24.5% [10]. Better study design and larger, more robust studies to fill the knowledge gap, including parent reports on child EBP, are warranted [11].

Although epidemiological studies have consistently identified different types of emotional and behavioral problems for boys and girls [12] there has been little research examining gender differences in child EBP in LMICs, including Nepal [13]. Most of the studies done in Nepal suggest higher rates of behavior problems in boys than in girls with boys having more externalizing problems and girls having more internalizing problems [10,14,15]. However, a broader, nationwide sample comprising children of all age groups is needed to confirm these findings.

It should be noted that in many LMICs, the population is multi-ethnic. However, few cross-cultural studies on child EBP problems have been performed in these countries, including Nepal. In order to explore possible within-country cultural differences, we decided to compare EBP between the seven largest castes and ethnic groups. Internationally, cross-cultural studies have shown that different cultural contexts might play a role in the prevalence and types of child mental problems as culture both defines and creates specific sources of distress [2,16].

Further, Nepal is a highly heterogeneous country not only culturally but also ecologically. However, little has been documented about EBP in children from different geographical locations and types of living area in Nepal. To capture a more nuanced picture of the distribution of EBP in the general child population, we also compared the amount of problems between the different geographic/ecological regions of the country and between different types of settlements/living areas.

**Aims of the present study:**

In this paper, we assessed the prevalence and amount (means) of parent-reported EBP in Nepali school-going children aged 6-18. To examine within-country diversity, we compared child problems between a) castes/ethnic groups, b) geographic regions, and c) types of living area. Finally, we looked for gender differences in EBP.

**Materials and Methods**

**Study site and population**

Nepal has a population of 28.6 million people (2019) and is topographically divided into three regions: The Himalaya (Mountain Region) to the North, the Middle Hills region, and the Tarai (the Southern flatland). There are 16 districts in the Mountain Region, 39 districts in the Middle Hills and 20 districts in the Tarai region. According to the Nepali demographic-social census [17], children below 18 years of age represent 44.4% of the total population: 22.5% boys and 21.9% girls. There are 126 different castes and ethnic groups. The term “caste” basically refers to a group of people who follow Hinduism. Hindu castes are ranked hierarchically in the following order of social status: (1) Brahmins (highest class), (2) Chhetri, (3) Vaishya, and (4) Sudra, also called Dalit (lowest class). The other ethnic groups in Nepal are indigenous nationalities and tribes, known collectively as the Janajati/Adivasi group. They have their own traditional cultures and specific languages, and do not necessarily adhere to, or fall under the Hindu caste system. According to the Census, the seven largest castes and ethnic groups are: Chhetri 16.6%, followed by Brahmin-Hill 12.2%, Magar 7.1%, Tharu 6.6%, Tamang 5.8%, Newar 5%, and Khas Kaami (the largest group of Dalits) 4.8% [17]. The Magar, Tharu, Tamang, and Newar all belong to the Janajati/Adivasi indigenous group.

**Study design**

The present study is a cross-sectional, cross-cultural epidemiological study in the general child population of Nepal.

**Subjects and procedure**

**Sampling method**

Based on the population distribution of the three main ecological/geographic regions of Nepal, we purposively selected three districts from the Mountain region, and six districts each from the Middle Hills and the Tarai regions. As our study includes an examination of child problems in different castes and ethnic groups, we wanted to ensure a high number of participants in each of these groups. Hence, the capital city Kathmandu(comprised of three districts) was added to the sample because of its multi-cultural population. In all, 16 districts were purposively selected from all over the country. Further, we selected four schools in each district (two government schools and two private schools) based on accessibility and referrals from schools - i.e. a total of 64 schools in the 16 districts. Students from grades 1 to 10 with six students in each grade (three boys and three girls), were then randomly selected using random number tables. Thus, in each district, 240 children were selected, which gave a total of 3840 children.

**Procedure**

Before commencing the study, ethical approval was obtained from the Ethical Board of the Nepal Health Research Council (NHRC) (ref. no. 1875; reg, no: 71/2017).

All school children aged 6–18 were eligible for the study, irrespective of their caste and ethnic background. However, schools for children with special needs and faith-based schools were excluded. Children’s caste and ethnic belongings were classified according to their parents’ own definition. Data collection work was monitored by the researcher (first author). Twenty trained research assistants and seven experienced supervisors performed the data collection work in the different districts. A meeting with the school management was conducted at each school and a written consent was obtained. An invitation letter was then sent to the parents and both oral and written information was provided. Informed consent was obtained from all participants. For illiterate parents, the research assistants verbally posed the questions to them and helped fill in the forms. Data were collected during September 2017–January 2018. Plotting of data was done manually during the first half of 2018 by three research assistants, supervised and controlled by the researcher. The overall participation rate was 99.5%. The proportion of missing items were not more than 0.1 % for any of the CBCL items.

**Measures**

Based on separate focus group conversations among teachers, parents and professionals about which screening tool would be the most appropriate for Nepal: the Achenbach System of Empirically Based Assessment (ASEBA) / Child Behavior Checklist (CBCL)/6-18 [18] or the Strength and Difficulties Questionnaire (SDQ 4-17) [19], we concluded that the ASEBA instruments would be the better instruments to use (i.e. for detecting problems, for clear and simple questions, and for higher cultural sensitivity).

**1. Child Behavior Checklist (CBCL/6-18)**

The CBCL has been translated to more than 100 languages and has established good psychometric properties across the world, including good criterion-related validity, good test-retest reliability, and good internal consistency as measured by Cronbach’s alpha [18]. It consists of 20 competence items and 113 problem items. The problem items are scored on eight syndrome scales, two broad-band subscales: Internalizing and Externalizing, and a Total Problems scale. The syndrome scales: Withdrawn/Depressed, Somatic Complaints and Anxious/Depressed together form the “Internalizing” scale and the scales: Rule-breaking Behavior and Aggressive Behavior together form the “Externalizing” scale. The Social Problems, Attention Problems and Thought Problems scales do not belong to either subscales but are included in the Total Problems scale, which is derived by summing up the individual item scores. The response format of questions on behaviors is: 0 = not true, 1 = somewhat or sometimes true, and 2 = very true or often true.

We used the Nepali version of the Child Behavior Checklist (CBCL)/6-18 that had been translated into Nepali in connection with a former Nepali study [10]. The teacher version (TRF) and the youth version (YSR) had both been validated and found acceptable for use in Nepal as reported in other studies [10,14]. In our study, we found an acceptable internal consistency for the parent version (CBCL) as indicated by Cronbach’s alphas for the eight syndrome scales: Withdrawn / Depressed: 0.71; Somatic Complaints: 0.79; Anxious / Depressed: 0.76; Rule-breaking Behavior: 0.76; Aggressive Behavior: 0.88; Social Problems: 0.73; Attention Problems: 0.80; Thought Problems: 0.75.

**2. Background information questionnaire**

The parents were asked to fill in a questionnaire asking about various background information data. In the present paper, we present the following selected variables: child gender, caste / ethnicity of the child, ecological / geographic region, types of living area, types of school, parents’ occupation, and parents’ educational level.

**Statistical analyses**

The ASEBA data management and SPSS statistics version 22.0 for Windows were used for all analyses. When computing the overall prevalence and mean estimates for Nepal, sampling weights were used to account for the oversampling for some regions and age-groups (i.e. according to the Nepal Census, 2011) [17]. We used Pearson’s chi square test for comparisons between groups on categorical variables. To assess group differences for continuous variables, analysis of variance (ANOVA) was done. For group comparisons involving more than three groups, post hoc comparisons were made using the Scheffé method. To indicate effect size, Hedges’ g was computed when comparing two groups. Partial eta squared was the selected effect size when more than two groups were compared. The significance level used in all tests was 0.005.

**Results**

**Background data**

Selected demographic background data are presented in Table 1.

As can be seen from the Table 1, boys and girls were almost equally distributed between the different castes and ethnic groups. The majority of participants, irrespective of caste / ethnic belonging, lived in semi-urban areas. Parents from the Tharu and Khas Kaami (Dalit) groups were the most illiterate, whereas the Newar group had the highest educational level. A substantial number of parents were migrant workers.

**Table 1. Distribution of selected demographic variables**

| **Caste and ethnic groups**  **N (%)** | **Chhetri**  866 (22.7) | **Hill Brahmin**  905 (23.7) | **Magar**  187 (4.9) | **Tharu**  246 (6.4) | **Tamang**  335 (8.8) | **Newar**  162 (4.2) | **Khas Kaami**  **(Hill Dalit)**  447 (11.7) |
| --- | --- | --- | --- | --- | --- | --- | --- |
| **Background variables** |  | | | | | | |
| ***Gender*** |  | | | | | | |
| Boys | 427 (49.3) | 457 (50.5) | 93 (49.7) | 124 (50.4) | 180 (53.7) | 68 (42.0) | 235 (52.6) |
| Girls | 439 (50.7) | 448 (49.5) | 94 (50.3) | 122 (49.6) | 155 (46.3) | 94 (58.0) | 212 (47.4) |
| ***Geographic Location*** |  | | | | | | |
| Mountain | 125 (14.4) | 158 (17.5) | 11 (5.9) | 1 (0.4) | 121 (36.1) | 6 (3.7) | 49 (11.0) |
| Hill | 431 (49.8) | 457 (50.5) | 128 (68.4) | 27 (11.0) | 116 (34.6) | 111 (68.5) | 265 (59.3) |
| Tarai | 310 (35.8) | 290 (32.0) | 48 (25.7) | 218 (88.6) | 98 (29.3) | 45 (27.8) | 133 (29.8) |
| ***Rural/Semi-urban/Urban*** |  | | | | | | |
| Rural | 227 (26.2) | 242 (26.7) | 28 (15.0) | 19 (7.7) | 111 (33.1) | 19 (11.7) | 124 (27.7) |
| Semi-urban | 423 (48.8) | 489 (54.0) | 117 (62.6) | 200 (81.3) | 181 (54.0) | 74 (45.7) | 259 (57.9) |
| Urban | 216 (24.9) | 174 (19.2) | 42 (22.5) | 27 (11.0) | 43 (12.8) | 69 (42.6) | 64 (14.3) |
| ***Types of School*** |  | | | | | | |
| Governmental | 359 (41.5) | 321 (35.5) | 91 (48.7) | 171 (69.5) | 195 (58.2) | 68 (42.0) | 348 (77.9) |
| Private | 507 (58.5) | 584 (64.5) | 96 (51.3) | 75 (30.5) | 140 (41.8) | 94 (58.0) | 99 (22.1) |
| ***Mother’s Occupation*** |  | | | | | | |
| Housewife | 596 (68.8) | 606 (67.0) | 134 (71.7) | 192 (78.0) | 237 (70.7) | 100 (61.7) | 319 (71.4) |
| Public Service | 53 (6.1) | 59 (6.5) | 6 (3.2) | 5 (2.0) | 14 (4.2) | 4 (2.5) | 0 (2.2) |
| Private Business | 69 (8.0) | 102 (11.3) | 20 (10.7) | 13 (5.3) | 24 (7.2) | 29 (17.9) | 36 (8.1) |
| Farmer | 116 (13.4) | 91 (10.1) | 16 (8.6) | 26 (10.6) | 39 (11.6) | 17 (10.5) | 58 (13.0) |
| Migrant Worker | 7 (0.8) | 13 (1.4) | 2 (1.1) | 3 (1.2) | 9 (2.7) | 2 (1.2) | 11 (2.5) |
| Others | 25 (2.9) | 34 (3.8) | 9 (4.8) | 7 (2.8) | 12 (3.6) | 10 (6.2) | 13 (2.9) |
| ***Father’s Occupation*** |  | | | | | | |
| Private business | 192 (22.2) | 266 (29.4) | 26 (13.9) | 54 (22.0) | 58 (17.3) | 68 (42.0) | 76 (17.0) |
| Farmer | 264 (30.5) | 251 (27.7) | 46 (24.6) | 88 (35.8) | 118 (35.2) | 31 (19.1) | 163 (36.5) |
| Migrant worker | 150 (17.3) | 131 (14.5) | 45 (24.1) | 28 (11.4) | 65 (19.4) | 20 (12.3) | 88 (19.7) |
| Others | 126 (14.5) | 120 (13.3) | 25 (13.4) | 65 (26.4) | 71 (21.2) | 28 (17.3) | 94 (21.0) |
| ***Family Education***^2^ |  | | | | | | |
| Illiterate | 83 (9.6) | 44 (4.9) | 10 (5.3) | 57 (23.2) | 47 (14.0) | 8 (4.9) | 76 (17.0) |
| Primary Level^3^ | 154 (17.8) | 147 (16.2) | 51 (27.3) | 71 (28.9) | 137 (40.9) | 26 (16.0) | 175 (39.1) |
| Secondary Level^4^ | 528 (61.0) | 544 (60.1) | 117 (62.6) | 110 (44.7) | 141 (42.1) | 83 (51.2) | 182 (40.7) |
| University Level^5^ | 101 (11.7) | 170 (18.8) | 9 (4.8) | 8 (3.3) | 10 (3.0) | 45 (27.8) | 14 (3.1) |

The place of residence (rural, semi-urban, urban) is based on the parents’ own reports and further verified by research assistants

^2^In the households with two parents, the higher education level was used

^3^ Primary level of education consists of grade 1 to 8

^4^ Secondary level of education consists of grade 9 to 12

^5^ University level includes Bachelor, Masters or PhD degree

**Prevalence of EBP for boys and girls –Total Sample**

In Table 2, we have presented prevalence as to the Achenbach classification of “normal”, “borderline” and “clinical” status according to American norms, both for the Total Problems scale and for the Externalizing and the Internalizing scales. Approximately, one fifth of all children had problems in the clinical range, with slightly more problems among boys. The prevalence of internalizing problems was higher than externalizing problems.

**Table 2**. **Prevalence of EBP for boys and girls –Total Sample**

|  | **Gender** | | Total |
| --- | --- | --- | --- |
|  | ***Male*** | ***Female*** |  |
| ***Total Problems T score*** |  |  |  |
| Normal (<60) | 68.7% | 71.5% | 70.1% |
| Borderline (60-63) | 11.2% | 10.4% | 10.8% |
| Clinical (>63) | 20.1% | 18.1% | 19.1% |
| ***Internalizing problems T score*** * |  |  |  |
| Normal (<60) | 61.9% | 66.9% | 64.4% |
| Borderline (60-63) | 12.7% | 10.3% | 11.5% |
| Clinical (>63) | 25.4% | 22.8% | 24.1% |
| ***Externalizing problems T score*** * |  |  |  |
| Normal (<60) | 76.5% | 80.3% | 78.4% |
| Borderline (60-63) | 7.9% | 6.8% | 7.4% |
| Clinical (>63) | 15.6% | 12.9% | 14.2% |

*P<0.05; **P<0.005; ***P<0.0005

**Adjusted prevalence for Nepal**

Since the Mountain region was somewhat over-sampled and the Middle Hills and Tarai regions under-sampled for 6-18-years-olds in our study, we computed sampling weights that took population numbers in the child population among the three geographic regions into consideration as well as the age distribution, both based on the Nepali 2011 census. As a result, the prevalence of CBCL Total Problems in Nepali 6-18-year-olds who scored in the clinical range was estimated to 18.3%; boys: 19.1% and girls: 17.6%.

**Prevalence of EBP between the different castes and ethnic groups**

The prevalence of child EBP varied among the different castes and ethnic groups (Table 3). It was highest for the low caste Khas Kaami group and lowest for the indigenous Tharu group

**Table 3. Prevalence of EBP between the different castes and ethnic groups**

|  | Chhetri | Brahmin- Hill | Magar | Tharu | Tamang | Newar | Khas Kaami | Total |
| --- | --- | --- | --- | --- | --- | --- | --- | --- |
| ***Total Problems Tscore*** *** |  | | | | | | | |
| Normal (<60) | 65.6 % | 71.8% | 73.3% | 78.9% | 72.5% | 70.4% | 63.3% | 69.5% |
| Borderline (60-63) | 12.1% | 9.5% | 8.6% | 8.1% | 13.1% | 13.6% | 9.6% | 10.7% |
| Clinical (>63) | 22.3% | 18.7% | 18.2% | 13.0% | 14.3% | 16.0% | 27.1% | 19.8% |
| ***Internalizing Problems Tscore*** *** |  | | | | | | | |
| Normal (<60) | 60.5% | 64.5% | 67.4% | 73.6% | 67.5% | 66.7% | 58.4% | 63.8% |
| Borderline (60-63) | 12.0% | 11.6% | 12.3% | 8.5% | 12.5% | 10.5% | 8.7% | 11.2% |
| Clinical (>63) | 27.5% | 23.9% | 20.3% | 17.9% | 20.0% | 22.8% | 32.9% | 25.0% |
| ***Externalizing Problems Tscore*** *** |  | | | | | | | |
| Normal (<60) | 76.2% | 77.3% | 81.3% | 88.2% | 82.4% | 78.4% | 72.0% | 77.9% |
| Borderline (60-63) | 6.6% | 8.4% | 7.0% | 4.9% | 7.2% | 8.0% | 6.9% | 7.2% |
| Clinical (>63) | 17.2% | 14.3% | 11.8% | 6.9% | 10.4% | 13.6% | 21.0% | 14.9% |

*P<0.05; **P<0.005; ***P<0.0005

**The amount of EBP for boys and girls – Total sample**

Table 4 shows mean problems for the whole sample by gender. Boys scored significantly higher than girls on Total Problems, Externalizing Problems, as well as on the three subscales: Social Problems, Thought Problems and Attention Problems. However, there were no gender differences in mean problems for the Internalizing scale. The effect sizes for the gender comparisons can be considered as small according to Cohen (1988) [20], with Hedges’ g ranging from 0.02 to 0.20.

**Table 4. The amount of EBP for boys and girls – Total sample**

|  | Boys  (N=1914)  Mean (SD) | Girls  (N=1906)  Mean (SD) | Total  (N= 3820)  Mean (SD) | Gender effect  F | Effect size  g ^a^ |
| --- | --- | --- | --- | --- | --- |
| Total Problems | 31.19 (26.67) | 28.14 (24.47) | 29.67(25.64) | 13.54 *** | 0.11 |
| Externalizing Problems | 8.44 (8.52) | 6.86 (7.35) | 7.65 (7.99) | 37.35 *** | 0.19 |
| Internalizing Problems | 9.01 (8.09) | 9.21 (7.96) | 9.11 (8.03) | 0.57 | -0.02 |
| Social Problems | 3.50 (3.25) | 3.18 (3.02) | 3.34 (3.14) | 9.92 ** | 0.10 |
| Thought Problems | 2.44 (3.07) | 2.11 (2.79) | 2.27 (2.94) | 12.17 *** | 0.11 |
| Attention Problems | 4.24 (3.75) | 3.52 (3.36) | 3.88 (3.58) | 38.62 *** | 0.20 |

*P<0.05; **P<0.005; ***P<0.0005; ^a^Hedges’ g

**The amount of EBP in the different castes and ethnic groups**

Table 5 presents the comparison of means on the different problem scales between the seven largest castes and ethnic groups using one-way ANOVA. The Khas Kaami (Dalit) group scored the highest and the Tharu group scored the lowest on all scales. In the post hoc multiple comparisons, the Tharu group differed from the Chhetri, the Brahmin Hill and the Khas Kaami groups on Total Problems, Externalizing Problems, Social Problems, Thought Problems and Attention Problems. For Internalizing Problems, the Tharu group differed only from the Chhetri and Khas Kaami, whereas the Tamang group differed from the Khas Kaami and Chhetri groups. However, the effect sizes were small, with partial eta squared ranging between 0.010 to 0.015.

**Table 5. The amount of EBP in the different castes and ethnic groups**

|  | Chhetri N =866  Mean (SD) | Brahmin-Hill  N = 905  Mean (SD) | Magar  N =187  Mean (SD) | Tharu  N =246  Mean (SD) | Tamang N = 335  Mean (SD) | Newar  N =162  Mean (SD) | Khas Kaami N = 447  Mean (SD) | Group effect  F | Partial Eta squared |
| --- | --- | --- | --- | --- | --- | --- | --- | --- | --- |
| Total problems | 32.67(27.55) | 29.95 (25.23) | 27.16 (25.65) | 22.53 (23.59) | 26.76 (22.21) | 28.82(22.38) | 34.15 (28.70) | 8.15*** | 0.015 |
| Externalizing Problems | 8.24 (8.55) | 7.78 (7.91) | 6.48 (7.08) | 5.69 (7.07) | 6.69 (7.36) | 7.82 (7.39) | 8.89 (8.77) | 6.04*** | 0.011 |
| Internalizing Problems | 10.18 (8.87) | 9.15 (7.78) | 8.48 (7.98) | 7.19 (7.50) | 8.04 (6.67) | 8.77 (7.25) | 10.40 (9.08) | 7.59*** | 0.014 |
| Social Problems | 3.71 (3.32) | 3.38 (3.06) | 3.14 (3.35) | 2.50 (2.77) | 3.02 (2.99) | 3.10 (2.71) | 3.77 (3.53) | 6.95*** | 0.013 |
| Thought Problems | 2.54 (3.09) | 2.24 (2.92) | 2.20 (3.04) | 1.58 (2.60) | 1.97 (2.44) | 2.12 (2.36) | 2.80 (3.47) | 6.38*** | 0.012 |
| Attention Problems | 4.22 (3.75) | 3.92 (3.62) | 3.77 (3.70) | 3.01 (3.18) | 3.66 (3.55) | 3.74 (3.32) | 4.38 (3.73) | 5.11*** | 0.010 |

*P<0.05; **P<0.005; ***P<0.000

**Associations between child EBP and geographic region and types of area**

There were no differences in the amount of problems between the three geographic regions, except for higher Internalizing Problems in the Mountain region. However, there were significant interactions between geographic regions and types of living area on Total Problems, as well as on the two broadband scales (Table 6). In the Mountain and Middle Hills regions, the problem scale scores were higher in the rural areas than in the semi-urban or urban areas. In contrast, children living in rural areas in the Tarai region scored lower than those living in semi-urban and urban areas. The sizes of the interaction effects were small, with partial eta squares less than 0.01.

**Table 6. CBCL scores by geographic regions and types of living area**

|  | **Total Problems**  Mean(SD) | **Internalizing Problems**  Mean(SD) | **Externalizing Problems**  Mean(SD) |
| --- | --- | --- | --- |
| **Mountain** |  | | |
| Rural Area (N=352) | 37.08(30.20) | 12.03(9.92) | 9.17(8.94) |
| Semi-Urban Area (N=134) | 22.82(21.30) | 7.60(6.91) | 5.19(6.23) |
| Urban Area (N=0) | - | - | - |
| Total (N=486) | 33.15(28.73) | 10.81(9.39) | 8.07(8.46) |
| **Hills** |  | | |
| Rural Area (N=460) | 32.92(28.12) | 9.87(8.68) | 8.61 (8.99) |
| Semi-Urban Area (N=902) | 29.46(23.73) | 9.14(7.62) | 7.62(7.34) |
| Urban Area (N=556) | 28.60(23.19) | 8.68(7.41) | 7.31(7.28) |
| Total (N=1918) | 30.04(24.75) | 9.18(7.84) | 7.77(7.76) |
| **Tarai** |  |  |  |
| Rural Area (N=58) | 20.05(20.99) | 5.69(6.62) | 5.55(6.22) |
| Semi-Urban Area (N=1117) | 26.93(25.83) | 8.27(7.72) | 7.08(8.10) |
| Urban Area (N=241) | 34.69(25.83) | 9.90(7.57) | 9.12(8.54) |
| Total(N=1416) | 27.97(25.59) | 8.44(7.69) | 7.36(8.15) |
| Main effect size of Geographic Region | F = 2.86  η^2^ = 0.002 | F = 6.81**  η^2^ = 0.004 | F = 1.12  η^2^ = 0.001 |
| Main effect size of Rural, Semi-Urban and Urban Areas | F=14.54***  η^2^ = 0.008 | F = 8.64***  η^2^ =0.005 | F = 10.63***  η^2^ = 0.006 |
| Effect of Interaction between Geographic Regions and Rural, Semi-Urban and Urban Areas | F = 13.85***  η^2^ = 0.011 | F= 12.94***  η^2^ = 0.010 | F = 10.014***  η^2^ = 0.008 |

*P<0.05; **P<0.005; ***P<0.0005; η^2^partial eta square

**Discussion**

This study assessed the prevalence and amount of EBP among school-going children in Nepal and compared the prevalence and amount of problems among different groups based on gender, caste/ethnicity, and types of area (urban, semi-urban and rural).

**Multicultural norms of the CBCL**

Based on data from 31 societies, Achenbach and Rescorla constructed different norm groups (high, medium, and low) for the CBCL, based on the omni-cultural mean of 22.5 (SD 5.6) that was found by averaging the Total Problem scores of the 31 cultures [21]. Nepal has not yet been included in the ranking of countries due to the lack of internationally published scientific studies. However, the present study, showing a Total Problems mean score of 29.7 (SD 25.6), suggests that Nepal should be placed under the high scoring countries.

**Comparison of results with other studies**

**Prevalence:**We found that the percentage of Nepali children who scored in the clinical range, i.e. above the American cut-off, was 19.1% for Total Problems, with an adjusted prevalence of 18.3%. Compared to the overall prevalence of mental health problems for schoolchildren in Asian countries as reported in an earlier review [4], the prevalence for Nepal seems high. However, the prevalence is consistent with findings from school studies in neighboring countries, e.g. China: 19.1% [9] and India: 23.3% [5].

The relatively high prevalence of child problems in these and other LMICs might be due to a higher level of environmental risk factors such as natural disasters [22, 23] and adverse social circumstances like poverty and child abuse and neglect [24, 25]. Social disadvantages and family fragmentation (e.g. caused by migrant working parents) are known to elevate level of stress affecting the mental health of parents as well as children [26, 27]. A possible explanation for the higher problem scores in Nepal could be the exposure to the devastating earthquake that hit the country in 2015 and the traumatic events that followed in its wake.The mental health effect of exposure to disasters like earthquakes, especiallyon children, is still largely unknown, and various resilience and posttraumatic growth (PTG) factors may be involved [28]. However, the present study was not designed to address a possible link between child EBP and disaster exposure, and other studies are needed to confirm the hypothesis. Further, the higher prevalence of EBP may be due to the poor and stressful living conditions experienced by many families in a LMIC country like Nepal. Yet another reason might be that Nepali parents might have a different threshold for reporting child EBP due to cultural norms. Cultural differences affecting parents’ ratings and interpretation of child behavior have been explored to a rather small extent internationally, and to our knowledge, no such studies have been performed in Nepal. Finally, it should be noted that the higher prevalence might be due to methodological reasons. In our study, we used a screening instrument (CBCL) and studies using screening instruments may yield higher prevalence rates than studies using diagnostic tools [4].

**Gender differences:**We found a higher level of Total Problems and Externalizing Problems in boys than in girls. This finding converges with findings from other international studies [12]. It also converges with the earlier Nepali dissertation study by Mahat mentioned in the introduction [10], suggesting the same gender pattern for Nepal. However, we did not find more Internalizing Problems in girls than in boys, contrasting to the findings from international meta-studies [12,29]. Our finding may be due to cultural or methodological factors and needs replication for verification. More studies on gender patterns in child EBP are warranted across cultures, especially from the less investigated LMICs.

**Types of problems:** An interesting finding in our study was the higher prevalence of internalizing problems (24.1%) compared to externalizing problems (14.2%). The finding converges with a recent epidemiological study from Kenya [30]. Like in the Kenyan study, a possible explanation for the elevated Internalizing Problems score could be the higher awareness and subsequent higher scorings of somatic symptoms by the parents. In Nepal, where there is very little awareness of mental problems in general, and particularly in children, parents tend to pay more attention to their child’s physical symptoms than their conduct and may define problems accordingly. Besides, Nepali children, like children from other South Asian countries, are socialized to control their frustrations and negative emotions, i.e. to internalize their problems, rather than acting them out [31]. Internationally, cross-cultural studies have shown significant variations in the relative dominance of internalizing versus externalizing problems [29, 32]. Due to different cultural norms and different socio-religious contexts, the types of problems that children express will differ. In countries where the culture discourages child aggression and other uncontrolled behaviors, internalizing problems like shyness, anxiety, and depression are noted more often, whereas in cultures that accept acting-out of emotions, externalizing behaviors are noted more often [29].

**Within-country differences in EBP**

**Castes and ethnic groups:**Cross-cultural comparisons showed that the Khas Kaami (Dalit) group had the highest prevalence of EBP. In Nepal, experiences of caste-based discrimination are found to be prevalent among the Dalit [33] and may be one of the main reasons for the higher problem level. In contrast, the Tharu, who mostly live in small villages in the Tarai region, showed the lowest amount of EBP. One possible explanation for the lower scores in this tribal, indigenous group may be that it has some strong protective factors, both at the individual, family, and social level. However, this finding needs further and more detailed investigation.

**Regions and types of living area:**We observed only small differences in the amount of child problems between geographic regions. However, some interesting interactions emerged for regions with types of living area. In the Mountain and Hills regions, children who lived in the rural areas had the highest problem scores, whereas in the Tarai region, children in the urban areas scored the highest. The higher problem scores in the rural areas in the Mountain region may be due to poverty and the tougher living conditions that exist in those areas [17]. The higher amount of problems found in the urban areas of the Tarai region may be due to the migration of families from the countryside to the cities in search of better working opportunities. Urbanization might lead to adjustment problems, more stress exposure, and increased vulnerability due to factors like overcrowding, low social support, inadequate security, and increased violence [34, 35]. The disparities in our findings converge with findings from the international literature. Some studies have found more child problems in rural areas [36], whereas others have found more problems in urban areas [34]. As countries differ in their economic development and cultural orientations, rural-urban differences in one country may not be generalized to other countries [37]. Based on the present study, we argue that this may be the case within a specific country as well –especially in countries as diverse as Nepal.

**Strengths and limitations of the study**

This epidemiological study is the first internationally published study on the prevalence and amount of EBP in Nepali schoolchildren as reported by their parents. We have used one of the most wide-spread and internationally validated instruments to assess child problems: the CBCL/6-18. The Cronbach’s alpha in our study was above 0.7 for all eight syndrome scales. We used sound methodology and thorough procedures in data collection, including helping illiterate parents with the filling in of forms and reaching out to parents who could not manage to visit the school. This probably increased parents’ trust and willingness to participate in the study and resulted in a very high participation rate: 99.5 %.

There are some methodological limitations of the study. First, our prevalence estimation was done by using the American norms of the CBCL as Nepali norms do not yet exist. Hopefully, future studies may provide separate norms for Nepal. Although we collected our data in the main geographic regions of Nepal and in 16 different districts in different parts of the country, we cannot claim that the results are representative for the whole country. Further, the number of participants in some of the castes and ethnic groups were small, which might have affected the results and made them less reliable. Hence, future studies with larger samples are recommended for those ethnic groups to confirm the findings.

Another limitation is that fathers’ reports were not assessed. Generally, fathers who are substantively involved in their children’s lives, may provide valuable information about their children’s problems [38].

The present paper focuses on the magnitude of child problems and how problems may vary across gender, different cultural groups, and types of living area. It does not include an examination of family- and social correlates of EBP. Such associations will be the focus in a subsequent paper currently in progress, and with the same sample of Nepali children.

**Conclusion**

The study provides new knowledge about the prevalence of child EBP in a LMIC. It amply demonstrates that in a country like Nepal, many children may suffer from various types of mental problems which may need attention. Furthermore, it highlights the importance of taking into account possible gender- and cultural differences in the amount and types of child problems, as well as pointing to rural – urban differences. The findings may be useful to the health authorities in developing child- and adolescent mental health services. Finally, the study provides important background information for both clinicians and teachers dealing with child mental health problems.

**Acknowledgements**

We are grateful to all participating parents and schools, and the team of data enumerators andsupervisors for making this study possible. Further, we would like to thank Dr. Arun Raj Kunwarand his child and adolescent psychiatry team at Kanti Children’s Hospital, Kathmandu, for their support.

**References**

1.	Letter-booklet-2020-world-population.pdf [Internet]. [cited 2021 Feb 19]. Available from: https://www.prb.org/wp-content/uploads/2020/07/letter-booklet-2020-world-population.pdf.

2.	Kieling C, Baker-Henningham H, Belfer M, Conti G, Ertem I, Omigbodun O, et al. Child and adolescent mental health worldwide: evidence for action. The Lancet. 2011 Oct 22;378(9801):1515–25.

3.	Erskine HE, Baxter AJ, Patton G, Moffitt TE, Patel V, Whiteford HA, et al. The global coverage of prevalence data for mental disorders in children and adolescents. Epidemiol Psychiatr Sci. 2017 Aug;26(4):395–402.

4.	Srinath S, Kandasamy P, Golhar TS. Epidemiology of child and adolescent mental health disorders in Asia. CurrOpin Psychiatry. 2010 Jul;23(4):330–6.

5.	Malhotra S, Patra BN. Prevalence of child and adolescent psychiatric disorders in India: a systematic review and meta-analysis. Child Adolesc Psychiatry Ment Health. 2014;8:22.

6.	Hossain MD, Ahmed HU, Chowdhury WA, Niessen LW, Alam DS. Mental disorders in Bangladesh: a systematic review. BMC Psychiatry. 2014 Jul 30;14(1):216.

7.	Malik TA, Siddiqui S, Mahmood A. Behavioural and emotional problems among school children in Pakistan: A telephonic survey for prevalence and risk factors. J Paediatr Child Health. 2019;55(12):1414–23.

8.	Mohammadi MR, Salmanian M, Ghanizadeh A, Alavi A, Malek A, Fathzadeh H, et al. Psychological problems of Iranian children and adolescents: parent report form of Strengths and Difficulties Questionnaire. J Ment Health Abingdon Engl. 2014 Dec;23(6):287–91.

9.	Qu Y, Jiang H, Zhang N, Wang D, Guo L. Prevalence of mental disorders in 6-16-year-old students in Sichuan province, China. Int J Environ Res Public Health. 2015 May 13;12(5):5090–107.

10.	Mahat P. A Study of the Prevalence and Pattern of Psychological Disturbances in School Going Children and Adolescents. PhD thesis. Fac Humanit Soc Sci Tribhuvan Univ. 2007.

11.	Chaulagain A, Kunwar A, Watts S, Guerrero APS, Skokauskas N. Child and adolescent mental health problems in Nepal: a scoping review. Int J Ment Health Syst. 2019;13:53.

12.	Rescorla L, Achenbach T, Ivanova MY, Dumenci L, Almqvist F, Bilenberg N, et al. Behavioral and Emotional Problems Reported by Parents of Children Ages 6 to 16 in 31 Societies. J EmotBehavDisord. 2007 Jul 1;15(3):130–42.

13.	Langer JA, Ramos JV, Ghimire L, Rai S, Kohrt BA, Burkey MD. Gender and Child Behavior Problems in Rural Nepal: Differential Expectations and Responses. Sci Rep [Internet]. 2019 May 21 [cited 2021 Feb 24];9. Available from: https://www.ncbi.nlm.nih.gov/pmc/articles/PMC6529428/

14.	Karki S, Laukkanen E, Länsimies-Antikainen H, Voutilainen A, Pietilä A-M. Self-reported emotional and behavioral problems in Nepalese adolescents from urban and rural areas. Nurs Health Sci. 2015 Mar;17(1):90–6.

15.	Adhikari RP, Upadhaya N, Gurung D, Luitel NP, Burkey MD, Kohrt BA, et al. Perceived behavioral problems of school aged children in rural Nepal: a qualitative study. Child Adolesc Psychiatry Ment Health. 2015;9:25.

16.	Canino G, Alegría M. Psychiatric diagnosis - is it universal or relative to culture? J Child Psychol Psychiatry. 2008 Mar;49(3):237–50.

17.	Nepal-Census-2011-Vol1.pdf [Internet]. [cited 2021 Feb 23]. Available from: https://unstats.un.org/unsd/demographic/sources/census/wphc/Nepal/Nepal-Census-2011-Vol1.pdf.

18.	Achenbach TM, Rescorla L. Manual for the ASEBA school-age forms & profiles: an integrated system of multi-informant assessment. 2001.

19.	Goodman R. The Strengths and Difficulties Questionnaire: a research note. J Child Psychol Psychiatry. 1997 Jul;38(5):581–6.

20.	Cohen J. Statistical Power Analysis for the Behavioral Sciences. [Internet]. Burlington: Elsevier Science; 2013 [cited 2020 Dec 27]. Available from: https://search.ebscohost.com/login.aspx?direct=true&scope=site&db=nlebk&db=nlabk&AN=923159.

21.	Achenbach TM, Rescorla L. Multicultural Supplement to the Manual for the ASEBA School-age Forms & Profiles: Child Behavior Checklist for Ages 6-18, Teacher’s Report Form, Youth Self-report : an Integrated System Fo Multi-informant Assessment. ASEBA; 2007. 108 p.

22.	Furr JM, Comer JS, Edmunds JM, Kendall PC. Disasters and youth: a meta-analytic examination of posttraumatic stress. J Consult Clin Psychol. 2010 Dec;78(6):765–80.

23.	John PB, Russell S, Russell PSS. The prevalence of posttraumatic stress disorder among children and adolescents affected by tsunami disaster in Tamil Nadu. Disaster Manag Response DMR Off PublEmerg Nurses Assoc. 2007 Mar;5(1):3–7.

24.	Patel V, Flisher AJ, Nikapota A, Malhotra S. Promoting child and adolescent mental health in low and middle income countries. J Child Psychol Psychiatry. 2008 Mar;49(3):313–34.

25.	Benjet C. Childhood adversities of populations living in low-income countries: prevalence, characteristics, and mental health consequences. CurrOpin Psychiatry. 2010 Jul;23(4):356–62.

26.	Hu H, Gao J, Jiang H, Jiang H, Guo S, Chen K, et al. A Comparative Study of Behavior Problems among Left-Behind Children, Migrant Children and Local Children. Int J Environ Res Public Health. 2018 Apr 1;15(4).

27.	Reiss F, Meyrose A-K, Otto C, Lampert T, Klasen F, Ravens-Sieberer U. Socioeconomic status, stressful life situations and mental health problems in children and adolescents: Results of the German BELLA cohort-study. PloS One. 2019;14(3):e0213700.

28. Calhoun LG, Tedeschi RG, editors. Handbook of posttraumatic growth: Research and practice. Routledge; 2014 Jul 10.

29.	Crijnen AA, Achenbach TM, Verhulst FC. Comparisons of problems reported by parents of children in 12 cultures: total problems, externalizing, and internalizing. J Am Acad Child Adolesc Psychiatry. 1997 Sep;36(9):1269–77.

30.	Magai DN, Malik JA, Koot HM. Emotional and Behavioral Problems in Children and Adolescents in Central Kenya. Child Psychiatry Hum Dev. 2018 Aug;49(4):659–71.

31.	Cole PM, Bruschi CJ, Tamang BL. Cultural differences in children’s emotional reactions to difficult situations. Child Dev. 2002 Jun;73(3):983–96.

32.	Weisz JR, Sigman M, Weiss B, Mosk J. Parent reports of behavioral and emotional problems among children in Kenya, Thailand, and the United States. Child Dev. 1993 Feb;64(1):98–109.

33.	Pariyar B, Lovett JC. Dalit identity in urban Pokhara, Nepal. Geoforum. 2016 Oct 1;75:134–47.

34.	Shahini M, Rescorla LA, Ahmeti AP, Begovac I, Dobrean A, Markovıć J, et al. Parent-reported behavioural and emotional problems in Albanian Kosovar children. Epidemiol Psychiatr Sci. 2015 Jun;24(3):266–73.

35.	Srivastava K. Urbanization and mental health. Ind Psychiatry J. 2009 Jul;18(2):75–6.

36.	Heflinger CA, Shaw V, Higa-McMillan C, Lunn L, Brannan AM. Patterns of Child Mental Health Service Delivery in a Public System: Rural Children and the Role of Rural Residence. J Behav Health Serv Res. 2015 Jul;42(3):292–309.

37.	Zhang H, Chang K, Zhang F, Greenberger E, Chen C. Mental health problems and coping styles of urban and rural high school students in China. J Community Psychol. 2011 Nov;39(8):1019–30.

38.	Jansen M, Bodden DHM, Muris P, van Doorn M, Granic I. Measuring Anxiety in Children: The Importance of Separate Mother and Father Reports. Child Youth Care Forum. 2017;46(5):643–59.
